# Partnerships to Improve Community Health: An Interview With Professor David Kindig of MATCH

**Published:** 2010-10-15

Professor David Kindig is emeritus professor of population and health sciences at the University of Wisconsin School of Medicine and Public Health and co-principal Investigator for the Mobilizing Action Toward Community Health (MATCH) project, which is funded by the Robert Wood Johnson Foundation. In this interview, Fran Kritz, editor of the Robert Wood Johnson Foundation Public Health page, talks with Dr Kindig about including the business sector in partnerships to improve community health.

**Figure:**
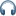
Partnerships to Improve Community Health (Part 1) (MP3–11Mb)

00:00 Fran Kritz I’m Fran Kritz, editor of the Robert Wood Johnson Foundation Public Health page. A new series of essays was commissioned for the current issue of *Preventing Chronic Disease*. The essays on partnerships to improve community health, were commissioned by the MATCH Project, Mobilizing Action Toward Community Health, which is based at the University of Wisconsin School of Medicine and Public Health and funded by the Robert Wood Johnson Foundation. We have the chance to speak by phone today with Professor Kindig, David Kindig, who is the emeritus professor of population and health sciences. Professor Kindig, thanks for being with us. Thanks for speaking with us today.
00:36 Dr David Kindig Thank you. I’m happy to be here.
Kritz We are talking today because there is a new set, a third set of essays in the current issue of *Preventing Chronic Disease*, and that set of essays is about partnerships toward improving community health. And I wondered if we could talk about that a bit. Why focus on partnerships? And what are some of the partnerships that the essays focus on?
00:59 Kindig Sure. Well, thanks for asking. We’ve actually had a whole set of essays that we commissioned a year ago and were presented in a meeting here in Madison last October, which have been profiled in other issues of *Preventing Chronic Disease*. And the way we kind of broadly, the way we sort of think about population health is in these 3 categories. The first was on metrics for measuring population health outcomes. How do we know how we’re doing? The second was on incentives. You know, what kinds of incentives can be used — financial and nonfinancial — at a community level or national level to sort of stimulate movement towards those outcomes? And then the third idea, which this current set of issues focuses on, is the partnerships that would be required both to bring those incentives into practice to move the outcomes in long- and in short-terms ways.
01:58 Kindig It really, it really Fran, it really builds on our population health thinking if it really, if population health is improved by investments and multiple determinants. Not just medical care. Not just public health, but the social environment — income, jobs, the physical environments, our behaviors, even genetics. Is it sort of implied? Nobody has responsibilities for all of those determinants and so it’s probably likely that a multisectoral solution involving partnerships is necessary to sort of reflect the way that health is produced in that broad way. So we invited these 8 essay writers to sort of focus on all different aspects of the partnership question and hopefully that’ll be useful both to practitioners and researchers in the field.
02:55 Kritz I was particularly interested in an essay by Andrew Weber, who is with the National Business Coalition on Health in Washington, DC. He focuses on ways in which the business community can improve community health. He gives several examples actually of community-business coalitions that have improved in one area, helping to educate the community about antibiotic resistance if there’s overuse of antibiotics; in another pushing for effort to greater fluoridation in order to improve oral health. Business is an interesting focus. Why that focus? How can that impact community health?
03:33 Kindig Right. I was really, I really wanted to get Andy Weber to write that paper and come to our conference because as you implied, often we don’t think of the business community as sort of a population health or a public health leader. Often they’re sort of left out of the equation, but in the broad multisectoral spirit that I mentioned earlier, it’s truly really very important and that the leadership and the resources that they could potentially bring to the table. One of the reasons that Andy’s paper is so important is that actually a lot of businesses have focused on wellness issues and issues of diet and exercise and putting health clubs into their factories and you know, worried about some of those kinds of issues.
04:27 Kritz What’s particularly important in this essay is Andy talks about the importance of employers being involved in some of the broader community investment issues beyond the workplace, so that they have a role not only in what they can do day-to-day in the workplace for safety or helping people with their behavior issues, but actually in the other issues in the community. We’ll actually start with number one, job creation, right? I mean that’s kind of what they’re all about and jobs produce income and income produces health, as we know. And in the education level in the community and we know how important — I mean they know that they need an educated workforce, but often they haven’t realized that also translates into a healthier workforce as well. And investments in the physical environment and what is well, all of those things. Bringing them to the table is really important.
05:23 Kritz The county health rankings of course, were released last February for the first time — a ranking of so many categories and every county in the country — and will be released again next February 2011. When you have heard feedback from the communities do any of them look to businesses as opportunities for partnership to help communities improve the health of some to many to more of their citizens?
05:48 Kindig Right. Well I think that’s a growing understanding. I mean, we tried in the rankings released, I mean we worked with state-partnered teams to help them help their communities and their counties to sort of understand, you know, how the rankings were coming down and you know, how they were going to be ranked. In many, we tried to create leadership teams and almost always with the leadership of the state public health departments, but in a number of places, reaching out to other partners from the education sector, from the business sector, from the medical community. You know, to sort of a) be aware of and hopefully b) in the future sort of be a part of that kind of solution.
06:32 Kindig You know, it’s been challenging often to draw the business community in. I mean, it’s been challenging here in Wisconsin, because we’ve had those rankings here for a number of years, even before this. But more and more, everybody, people are realizing that public health is everybody’s problem and we have to draw these other stakeholders to the table and more and more they’re interested. Actually, Andy Weber, you’ll see in the future on the MATCH website, Andy Weber is actually doing a few case studies for us where they’re actually profiling businesses that have either made location or relocation decisions based on the community health data of measures such as we find in the county health rankings. So I believe it’s going to become increasingly important.
07:22 Kritz I found all of the essays interesting. There were a variety of different kinds of partnerships — government, of course, and health care organizations working together. All of the essays, as you mentioned, can be read in the current issue of *Preventing Chronic Disease* and I invited our listeners to read through all of them. But I wonder if you would focus on a couple of more partnership ideas that have been presented by the essay writers.
07:46 Kindig Sure. I’m happy to do that and if we had an hour, I’d be happy to sort of give you a little mini-lecture on all of them. So let me just highlight a few. There was one by Professor Fawcett and his colleagues in Kansas and they actually focused on some broad ideas about what makes partnerships work and not work. And actually in that essay, he has a whole set of 7 recommendations about strengthening collaborative partnerships from monitoring systems, actions plans, an adequate funding base, technical assistance, a whole variety of things.
08:24 Kindig So people interested in that could take a look at that essay and even quickly just look at those 7 to get some idea. And in another sense, and there’s a really wonderful long essay by Professor Mays at the University of Arkansas, who is one of the nation’s experts, world’s experts, on public health partnerships and a lot of useful information about how the public health community can take a lead on having this happen.
08:53 Kritz Professor David Kindig, emeritus professor of population and health sciences at the University of Wisconsin School of Medicine and Public Health and also, co-principal Investigator for the MATCH, Mobilizing Action Toward Community Health project, which is funded by the Robert Wood Johnson Foundation. Thanks so much for speaking with us.
Kindig I’m happy to have done it and we’d be happy to hear any comments that people have about those essays.
09:19 Kritz I’m Fran Kritz for the Robert Wood Johnson Foundation.

**Figure:**
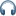
Partnerships to Improve Community Health (Part 2) (MP3–3.5Mb)

00:00:00 Fran Kritz I’m Fran Kritz, editor of the Robert Wood Johnson Foundation Public Health page. What’s next for the county health rankings? We recently asked that question of David Kindig, co-principal investigator of the MATCH Project, Mobilizing Action Toward Community Health, which is based at the University of Wisconsin and funded by the Robert Wood Johnson Foundation. MATCH released the first set of county health rankings — health parameters measured in every county of the country — and will re-release the updated measures in February 2011. Here’s what he had to say.
00:33 Dr David Kindig The MATCH acronym of our project with Robert Wood Johnson stands for Mobilizing Action Towards Community Health. And the rankings are hopefully the mobilization of getting people aware of how they rank and where their opportunities for improvement are. But you know, that, in some ways, is the easy part. Don’t tell my colleagues who have to do the rankings that because it’s hard work here at our institute. But how do you know, once you’ve mobilized action, you know, what do you do? You know, to sort of move these, move these outcomes and move these metrics. And that’s the work that’s going on, going on in the future and we have a tag, “take action,” part of that website but I think that the attention — these have raised attention in the public and private sector, in foundations and in CDC. And even on Friday, the Surgeon General talked about the release of the National Public Health and Prevention Strategy that’s going to roll out by March. And so, there’s lots going on, you know, beyond here but we don’t yet have the answers. We know how to measure the outcomes pretty well, I mean there’s ways we can do better on that but, but the whole thing about finding the right kind of financial and nonfinancial incentives, which was last month, and partnerships across sectors to pull it out, to make it happen.
02:00 Kindig I mean, this is still a challenge for us and you know, one of the issues around partnerships is, for these kind of tough outcomes, you know, there’s not a single entity that’s accountable. That’s one of the problems, you know? Different sectors have different pieces of the action; the government, the private sector, businesses, schools, whatever. Actually put in a plug for my blog, improvingpopulationhealth.org, and I’ve blogged twice on this one, about who is accountable? Which is really a partnership kind of a blog. And then another one about, on, do we need a super integrator? Which is sort of an idea about pulling all of these partnerships together, so any readers might want to check that out as well. So you’re right, there’s a lot to be done in terms of making movement. But I think we’re under way and I tell you, I’ve been slogging in this pop health field for a while and the last 5 or 6 years it’s a little bit of a different story than it was 10 or 15 years ago. So that’s hopeful.

